# The COVID-19 Own Risk Appraisal Scale (CORAS): Development and validation in two samples from the United Kingdom

**DOI:** 10.1177/1359105320967429

**Published:** 2022-03

**Authors:** Rusi Jaspal, Emanuele Fino, Glynis M. Breakwell

**Affiliations:** 1 Nottingham Trent University, UK; 2 Imperial College, UK

**Keywords:** COVID-19, COVID-19 Own Risk Appraisal Scale, COVID-19 prevention, fear of COVID-19, perceived risk, scale validation

## Abstract

Perceived risk is an important determinant of the adoption of preventive behaviours. In this article, the psychometric properties of the COVID-19 Own Risk Appraisal Scale (CORAS), including its development and validation in two samples in the United Kingdom, are described. The CORAS is a measure of perceived personal risk of contracting the disease, incorporating primarily intuitive with some analytic risk estimates. Exploratory and confirmatory factor analyses were performed on data from 470 participants in the United Kingdom who completed the CORAS, the Fear of COVID-19 Scale and the COVID-19 Preventive Behaviours Index. Results showed that a unidimensional, six-item model fits the data well, with satisfactory fit indices, internal consistency and high item loadings onto the factor. We found no statistically significant differences by age, gender or ethnicity. The CORAS correlated positively with the Fear of COVID-19 Scale and the COVID-19 Preventive Behaviours Index, suggesting good concurrent validity.

## Introduction

Following its initial clinical observations in Wuhan, China in December 2019, COVID-19 has rapidly spread globally. Many countries instigated a nationwide lockdown at least during the early phases of the outbreak. Subsequently lockdowns were eased, only to be reintroduced again locally where infection rates spiked. Fear and uncertainty about COVID-19 remain high. There is, currently, no effective cure for COVID-19, no vaccine against it and future surges of the virus are predicted. Clearly the so-called social distancing policies, are an important aspect of COVID-19 prevention (Michie et al., 2020). However, adherence to these policies and to other COVID-19 preventive measures, such as testing and the uptake of a future vaccine, will depend, in part, on people’s perception of their own personal risk of infection. There has been a resurgence of COVID-19, following the initial outbreaks, and there remains a risk of additional outbreaks in the future. It is important to develop a robust, reliable and valid measure of perceived own risk, as this is particularly likely to influence cognitions, emotions and action in relation to the pandemic, its prevention and its management. This article describes the development, validation and psychometric properties of the COVID-19 Own Risk Appraisal Scale (CORAS).

### Risk perception

Assessment of risk involves examining two discrete facets: the likelihood that the bad thing (the hazard) will happen and the severity of the effects of the bad thing when it does happen. However, risk perception cannot be reduced to any simple subjective correlate of an estimate of risk based on the product of probability and consequences. Many factors have been shown to affect how individuals and groups perceive specific hazards. The risk perception process can be influenced by socio-demographic characteristics; past experience; personality traits; emotional state; ideological and belief systems; identity processes; and many other factors (Breakwell, 2014). It is also influenced by social representations of the hazard created communally (Sammut et al., 2015) and by processes of social amplification and attenuation, for instance, through political policy and mass media reporting (Pidgeon et al., 2003).

Given the variety of influences working upon it, it is hardly surprising that individuals and groups differ in their perception of risk. However, these differences matter because risk perceptions associated with a hazard affect thoughts, feelings and behaviour concerning that hazard. Particularly in relation to health hazards, perceived risk has been shown to influence behaviour (Ferrer and Klein, 2015). Since the way perceived risk affects behaviour varies across individuals, it is important to examine those effects. To do so, it is vital to have valid and reliable instruments for indexing risk perception.

In developing such instruments, it is important to distinguish, and not conflate, various elements in the perception of the hazard. First, the distinction between perceived likelihood of harm to oneself or to others. Second, the distinction between the perceived extent of the potential harm to oneself or to others. Third, the distinction between the appraisal of likelihood or extent of harm and the emotions aroused by that appraisal. Fourth, the distinction between an informed or rational (analytical) appraisal of the likelihood or extent of harm and an (intuitive) appraisal based on habit, preconception or having a ‘sense’ of being at risk. This final distinction is akin to the distinction between fast and slow thinking made by Kahneman (2011). The purpose of the scale described here was to index appraisal of the likelihood of one’s own infection with COVID-19. The scale items focus primarily upon the intuitive appraisal of that risk.

### Measuring perceived risk of COVID-19

It has been recognised that an index of perceived risk is needed for COVID-19. It will facilitate modelling of psychological and behavioural responses to the pandemic and to its consequences. Thus far, most empirical studies that have attempted to index COVID-19 risk perception have used single-item measures. In their United Kingdom study, Harper et al. (2020) simply asked participants to self-report whether they considered themselves at ‘low-’, ‘medium-’ or ‘high-risk’. Kim et al. (2020) used ‘Do you think you have the same risk as others?’ to measure perceived risk, allowing only a yes/no response. Given the multidimensional nature of risk perception, it is limiting to utilise single-item measures which may not capture adequately cognitive and affective dimensions of perceived personal risk of COVID-19. In their Korean study, Lee and You (2020) used two separate items – one focusing on the perceived possibility of infection and the other on the perceived severity of infection. This two-item measure has the advantage of capturing both facets of risk.

Various scales have been developed to capture the emotional concomitants of COVID-19, including the Fear of COVID-19 Scale (Ahorsu et al., 2020), the Coronavirus Anxiety Scale (Lee, 2020), and the COVID Stress Scales (Taylor et al., 2020). Although these scales often correlate positively with measures of perceived risk of COVID-19, partly because extant measures of perceived risk tend to include items focusing on emotion, they do represent distinct constructs. While fear, anxiety and stress have variable relations with behaviour change and action to reduce one’s risk (Harper et al., 2020; Witte and Allen, 2000), perceived risk of infection has been shown to be a more robust predictor of this important dependent variable. It is therefore important to differentiate between the emotional concomitants of COVID-19 and the perceived likelihood of contracting the disease and a scale which focuses specifically on perception of one’s own risk of COVID-19 is needed.

Yıldırim and Güler (2020) developed the 8-item COVID-19 Perceived Risk Scale (CPRS) to assess perceived risk of infection. They amended the 8-item SARS Risk Perception Scale (Brug et al., 2004) and, like it, theirs included a cognitive dimension (focusing on the perceived likelihood of COVID-19 infection) and an emotional dimension (focusing on worry about *significant others* contracting the virus). They suggest that the scale can be used as an overall measure of perceived risk or that its subscales can be used separately to measure the analytic and emotional dimensions of perceived risk. Another study used items from previous studies to construct a measure of ‘COVID-19 risk perception’ (Dryhurst et al. (2020). This scale exhibited variable internal reliability across different countries where it was developed (alpha between .60 & .82) and no factor analysis was reported to demonstrate the psychometric properties of the scale. Like the CPRS, the scale did not focus exclusively on perceived *personal* risk and included items tapping into fear, besides perceived risk, of COVID-19.

### The COVID-19 Own Risk Appraisal Scale

There is a need for an index of perceived risk which does not conflate perceived risk of COVID-19 to others and one’s *own* perceived risk since perceived risk to others might not necessarily guide behaviour in response to the pandemic. It is also important to discriminate between the emotional and analytic dimensions of risk perception. Accordingly, we developed the COVID-19 Own Risk Appraisal Scale (CORAS) as a measure of perceived personal risk of contracting the disease. The Perceived Risk of HIV Scale (Napper et al., 2012) was deemed to be a useful basis for the development of the CORAS partly because, like HIV, risk of COVID-19 infection can be influenced by modifications to one’s behaviour. Both scales focus on estimates of own risk, rather than risk of others. Furthermore, as with HIV, there is emerging evidence that people think about their risk of COVID-19 infection in both analytical and intuitive ways (Breakwell and Jaspal, 2020). Consequently, CORAS was designed to offer an index of one’s own risk appraisal that included both analytical and intuitive items. The CORAS’ specific emphasis on perceived likelihood of own infection is a valuable addition to the other scales that focus upon emotional reactions to the hazard.

## Methods

### Ethics

The study received ethics approval from Nottingham Trent University’s College of Business, Law and Social Sciences Research Ethics Committee (ref: 2020/214).

### Participants

A sample of 479 individuals was recruited, of whom 470 provided complete demographic data and answered all other questions in the study and were included in the analyses. Three hundred and three (64.5%) were female, 165 (35.1%) were male, and 2 were gender non-binary (0.43%). Participants were aged between 18 and 72 years (*M* = 32, *SD* = 12) and came from various ethnic and socio-demographic backgrounds. There was an even distribution of White British and Black, Asian and Minority Ethnic (BAME) participants in the sample. Table 1 includes detailed information on the social and demographic characteristics of participants.

**Table 1.** Socio-demographic characteristics of participants.

Variables	Total (*N* = 470)	Females (*N* = 303)	Males (*N* = 165)	Non-binary (*N* = 2)	*p* ^a^
Age (years)					
*M* (SD)	32.7 (12.4)	32.6 (12.0)	32.9 (13.0)	21.0 (4.2)	0.399
Ethnicity (detailed)					
*N* (%)					0.179
White British	243 (52)	169 (56)	74 (45)	0 (0)	
White and Black Caribbean	4 (1)	3 (1)	1 (1)	0 (0)	
White and Asian	5 (1)	3 (1)	2 (1)	0 (0)	
White Other	2 (0)	0 (0)	2 (1)	0 (0)	
Pakistani	57 (12)	34 (11)	22 (13)	1 (50)	
Bangladeshi	15 (3)	7 (2)	8 (5)	0 (0)	
Indian	68 (15)	40 (13)	28 (17)	0 (0)	
Caribbean	28 (6)	18 (6)	9 (5)	1 (50)	
African	48 (10)	29 (10)	19 (12)	0 (0)	
Ethnicity (main)					
*N* (%)					0.027
White British	243 (52)	169 (56)	74 (45)	0 (0)	
BAME	227 (48)	134 (44)	91 (55)	2 (100)	
Qualification					
*N* (%)					0.232
High school (GCSE/O-Levels)	48 (10)	21 (7)	26 (16)	1 (50)	
High school (AS/A-Levels)	139 (30)	93 (31)	46 (28)	0 (0)	
Undergraduate	197 (42)	134 (44)	62 (38)	1 (50)	
Postgraduate	73 (16)	45 (15)	28 (17)	0 (0)	
Apprenticeship	5 (1)	4 (1)	1 (0)	0 (0)	
Other	7 (1)	5 (2)	2 (1)	0 (0)	
None	1 (0)	1 (0)	0 (0)	0 (0)	
Employment					
*N* (%)					0.708
Employed	239 (51)	157 (52)	82 (50)	0 (0)	
Self-employed	37 (8)	22 (7)	15 (9)	0 (0)	
Furloughed	31 (7)	19 (6)	12 (7)	0 (0)	
Student	114 (24)	72 (24)	40 (24)	2 (100)	
Retired	10 (2)	7 (2)	3 (2)	0 (0)	
Unemployed	39 (8)	26 (9)	13 (8)	0 (0)	

a Results from parametric bivariate tests of significance (*t*-test or ANOVA where appropriate for continuous variables and *χ*^2^ test of independence for categorical variables).

### Design and procedure

Participants were recruited at two points during the outbreak in the United Kingdom – on 8 July (*N* = 251) and on 14 August 2020 (*N* = 228). All participants had to be at least 18 years old and fluent in English to participate in the study. They were all recruited on *Prolific*, an online participant recruitment platform, where they were invited to participate in a cross-sectional survey study of their perceived risk and self-protection behaviour in response to the COVID-19 pandemic in the United Kingdom. Each participant was debriefed, signposted to available support and counselling services in the United Kingdom should they wish to use them, and paid £1.75 for their time.

### Measures

#### Perceived risk of COVID-19

The COVID-19 Own Risk Appraisal Scale (CORAS) was created by adapting the 10-item Perceived Risk of HIV Scale (Napper et al., 2012) and selecting nine items which were relevant to COVID-19. To identify the pool of the nine CORAS items, we evaluated whether each of the items from the original measure represented a good fit and could successfully be adapted to measure perceived risk of COVID-19. This process was informed by two main criteria: (1) item performance data from the original study on perceived HIV risk and (2) expert evaluation of the item’s content. Members of the research team have expertise in risk perception, HIV risk and scale development. The nine items selected for the CORAS included: ‘I am sure I will NOT get infected with COVID-19’ and ‘I feel vulnerable to COVID-19 infection’. All items were scored on a 5-point ordinal scale (please see the appendix for a detailed presentation of the items and response options). The CORAS total scores were computed by summing up the scores of individual items, with higher scores indicating higher perceived risk of COVID-19.

#### Fear of COVID-19

The Fear of COVID-19 Scale (Ahorsu et al., 2020) was adapted to measure fear of COVID-19. The adapted scale included 10 items and was measured on a 5-point scale (1 = strongly disagree and 5 = strongly agree). Items included ‘I do not worry much about COVID-19’ and ‘When I think about COVID-19, my heart races and palpitates’. A higher score indicated greater fear of COVID-19. Cronbach’s alpha analyses from the original study showed that the scale was internally consistent (0.82), which was consistent with results observed in the current study (0.83, *N* = 470)

#### COVID-19 preventive behaviours index

The COVID-19 Preventive Behaviours Index (Breakwell et al., 2020) was used to measure the likelihood of engaging in specific behaviours that can decrease one’s risk of exposure to COVID-19. The scale consisted of 10 items and was measured on a 5-point scale (1 = Items included ‘How likely is it that, during the COVID-19 outbreak you will keep a distance of 2 m in your everyday interactions with people outside of your household?’ and ‘. . .use a facemask when you leave your home?’ (1 = extremely unlikely and 5 = extremely likely). A higher score indicated greater intention to engage in preventive behaviours. In the current study, the index showed satisfactory internal consistency (Cronbach’s alpha = 0.76, *N* = 470).

### Statistical analyses

We used Exploratory Factor Analysis (EFA) to explore the dimensionality and factor structure of the CORAS and Confirmatory Factor Analysis (CFA) to test the model derived from EFA. We used Item Response Theory (IRT) to examine items’ parameters, information, and differential item functioning.

EFA is a statistical technique that is used to reduce a set of observed variables to a smaller number of underlying ‘latent’ dimensions (factors). The input of EFA is a correlation matrix computed across a set of observed variables, whereas the main output consists of a newly determined matrix, holding correlations between the observed variables and a smaller number of latent factors (Costello and Osborne, 2005). Assuming our data to be ordinal, we first extracted the polychoric correlation matrix from the data and used it to perform EFA with Weighted Least Square estimation (Schmitt, 2011). We used four criteria to assess the dimensionality of the CORAS and determine the number of factors to retain: (i) Parallel analysis (Horn, 1965), (ii) the Very Simple Structure method (Revelle and Rocklin, 1979), (iii) Velicer’s (1976) Minimum Average Partial test, and (iv) the internal consistency and interpretability of the solution.

Parallel analysis compares eigenvalues from the observed correlation matrix to the corresponding eigenvalues from a correlation matrix estimated on randomly generated data, assuming equal sample size between the empirical and the simulated datasets. Eigenvalues represent the amount of variance explained by each factor. We plotted results on a scree test to identify factors with observed eigenvalues greater than those obtained at random, considered to be candidates for retention. The Very Simple Structure method compares a range of empirical solutions to a simplified solution obtained by freely estimating the top-loading item per factor and constraining the other items’ loadings to zero, iteratively on alternative *n*-factor solutions. The factor solution that maximises the fit of the simplified pattern matrix to the original observed matrix is a valid candidate for retention. The Minimal Average Partial test aims to determine the item correlation matrix that produces the best solution, namely the one that maximises systematic variance and minimises residual variance. It compares a set of average squared correlations, each estimated by progressively partialing out factors, from 1 to *k* − 1 (*k* represents the total number of items). The lowest squared partial correlation indicates the optimal solution. Finally, we used Cronbach’s alpha to assess the internal consistency of the CORAS.

Next, we tested the retained model by means of CFA. This is a statistical technique used to test the fit of a theoretical model of relations between observed variables and latent factors to a set of empirical observations. It requires the speciﬁcation of a measurement model, the fit of the model to the data, the assessment of the model’s ﬁt, and the interpretation of the model. We used the WLSMV estimator with robust standard errors (Muthén, 1983) to fit the model, and McDonald’s (1999) Omega to assess the reliability of the model (Green and Yang, 2009).

We then used IRT to estimate items’ discrimination and information, and differential functioning. IRT is a family of statistical models that rely on the estimation of the probability to endorse an item response as a function of the respondent’s positioning on a hypothesised latent dimension (theta; van der Linden and Hambleton, 1997). We estimated and examined item parameters (slopes, *α* and response category threshold parameters, *β*), and item information functions (IIFs). Slopes represent the ability of items to discriminate respondents on the theta continuum. In the specific case of the CORAS, ‘threshold parameters represent the level of the perceived risk necessary for a participant to respond above a threshold category with a 0.50 probability’ (Napper et al., 2012, p. 1078), whereas item information represent the item’s contribution in terms of statistical information over a range of scores on the latent dimension. We used the Graded Response Model (GRM) for polytomous data (Samejima, 1969) and examined the model by evaluating Item Response Categories Characteristic Curves (IRCCs), Item Information Curves (IICs) and Test Information Curves, respectively.

GRM assumes that the probability of selecting a higher response category increases as the perceived risk level increases. Therefore, participants with higher perceived risk are assumed to select higher item response categories than participants with lower perceived risk, and vice versa. Prior to fitting the model, we assessed two fundamental assumptions: (i) unidimensionality and (ii) item local independence. We assessed unidimensionality by examining results from EFA, and local independence by examining items’ residual correlations, looking at correlations greater than the absolute average residual correlation +0.20 as indicators of local dependence. We then tested for differential item functioning by respondents’ socio-demographic characteristics, using ordinal logistic regression/IRT (Choi et al., 2011). To do so, we recoded gender (females, males), age (<30 years, ⩾30 years) and main ethnicity (White British, BAME) into categorical variables when required, and tested for differential item functioning by applying the *χ*^2^ test (alpha = 0.01), the pseudo *R*^2^ change (significant change=0.02), and the proportional *β* change (significant change = 0.1) detection criteria (Choi et al., 2011). Finally, we tested for the concurrent validity and the criterion validity of the CORAS by estimating Spearman’s correlations between total CORAS scores and total Fear of COVID-19 scores, and between total CORAS scores and total scores of the COVID-19 Preventive Behaviours Index, respectively.

The analyses were conducted on two equally sized, randomly selected sub-samples. Specifically, we explored the factor structure and dimensionality of the CORAS using the first sub-sample (*N* = 235), whereas we ran CFA and IRT analyses on the second sub-sample (*N* = 235). Both sub-samples reflected the overall distribution of data, displaying similar characteristics in terms of gender, age, and ethnicity. However, routine data screening and validity analyses were conducted on the full sample (*N* = 470).

All analyses were performed by using the statistical programming language R (Version 3.6.2) (R Core Team, 2020) and the following packages: psych (Revelle, 2020) for EFA, lavaan (Rosseel, 2012) for CFA, semTools (Jorgensen et al., 2020) for reliability, and mirt (Chalmers, 2012) for IRT, lordif for differential item functioning (Choi et al., 2011). Tables were produced with the auxilium of the furniture package (Barrett and Brignone, 2017).

## Results

### Preliminary data screening

First, we screened responses for missing values and unengaged patterns (*SD* < 0.3), and we examined the distribution of data. We found no unengaged responses, and variables displayed values of skewness and kurtosis within the range of ±2. Correlations between the CORAS items were all significant (*p* < 0.001), ranging from low to high (Spearman’s rho = 0.23–0.77). Items’ descriptive statistics and correlations are presented in Table 2.

**Table 2.** CORAS correlation matrix (*N* = 470).

Item number	*M*	*SD*	1	2	3	4	5	6	7	8	9
1. Gut feeling of own likelihood of infection	2.60	0.82									
2. Can picture self catching it	2.83	0.93	0.44								
3. Sure I will not be infected	3.59	1.12	0.56	0.44							
4. Unlikely to get infected	3.03	1.13	0.68	0.42	0.57						
5. Feel vulnerable	2.70	1.14	0.52	0.42	0.43	0.50					
6. Self-rated chance of infection	3.17	0.71	0.57	0.41	0.49	0.52	0.43				
7. Worry about getting infected^a^	2.99	1.25	0.48	0.33	0.41	0.42	0.60	0.39			
8. Concern about getting infected^a^	2.74	1.15	0.42	0.37	0.38	0.38	0.58	0.42	0.77		
9. Chance of getting infected^a^	4.13	0.98	0.36	0.35	0.48	0.32	0.27	0.36	0.27	0.23	

M and *SD* represent mean and standard deviation, respectively. All correlations are expressed as Spearman’s rho values. All correlations are statistically significant at *p* < 0.001.

a Not included in the final version of the scale.

### Exploratory factor analysis

**Figure 1.**
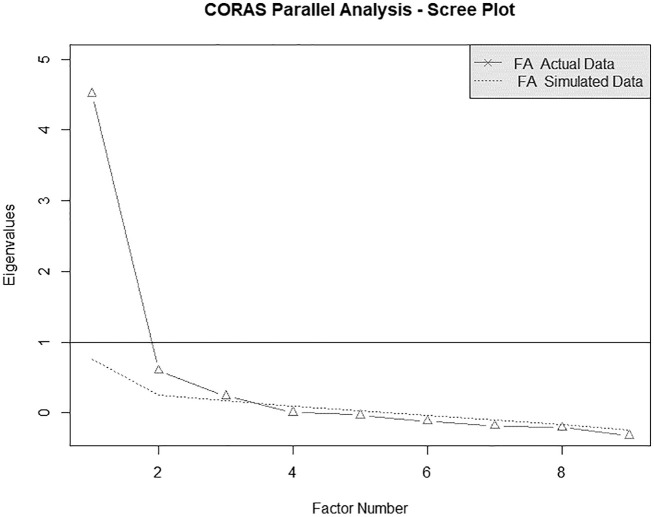
Exploratory factor analysis, scree plot.

We extracted the polychoric correlation matrix from the first randomly selected sub-sample (*N*=235) and ran EFA (TLI=0.93, RMSEA = 0.20 with 90% CI = 0.18–0.22, BIC = 128.63). We assessed the dimensionality of the CORAS by means of parallel analysis. The scree plot showed three empirically extracted factors with eigenvalues greater than the eigenvalues of corresponding factors from the random data set, although only the first factor showed an empirical eigenvalue greater than one (4.51 vs 0.60 extracted on the second factor) (Figure 1). Moreover, the exploration of the three-factor solution showed several items’ cross-loadings, with the third factor being not internally consistent, leading to a poorly interpretable model.

To further evaluate the scale’s dimensionality, we considered results from the Very Simple Structure and Minimal Average Partial analyses. The Very Simple Structure complexity goodness-of-fit index achieved a maximum of 0.89 with one factor, and similarly, the Minimal Average Partial coefficient achieved a minimum of 0.06 with one factor. The manual inspection of the pattern matrix obtained from the one-factor and the unrotated two-factor solutions, respectively, allowed us to confirm that the former was the most theoretically interpretable, with all items loading highly (⩾0.53) onto a single factor (total variance explained = 50%), and the measure being internally consistent (Cronbach’s alpha = 0.87, with 95% CI = 0.84–0.89), with no item found to increase alpha if deleted. Table 3 reports the pattern matrix from the one-factor and the three-factor solutions.

**Table 3.** CORAS exploratory factor analysis.

Item number	One-factor solution			Three-factor solution				
	F1	Item communality	Item variance	F1	F2	F3	Item communality	Item variance
1. Gut feeling of own likelihood of infection	0.79	0.63	0.37	0.80	0.05	0.04	0.73	0.27
2. Can picture self catching it	0.61	0.37	0.63	0.07	0.15	0.54	0.45	0.55
3. Sure I will not be infected	0.75	0.56	0.44	0.45	0.05	0.38	0.60	0.40
4. Unlikely to get infected	0.77	0.59	0.41	0.90	0.00	−0.04	0.77	0.23
5. Feel vulnerable	0.77	0.59	0.41	0.21	0.56	0.13	0.61	0.39
6. Self-rated chance of infection	0.69	0.48	0.52	0.30	0.10	0.43	0.51	0.49
7. Worry about getting infected^a^	0.73	0.53	0.47	0.09	0.81	0.00	0.75	0.25
8. Concern about getting infected^a^	0.71	0.50	0.50	−0.07	0.99	−0.02	0.90	0.10
9. Chance of getting infected^a^	0.53	0.28	0.72	−0.06	−0.03	0.82	0.59	0.41
Total variance explained	50%			24%	25%	17%		
Cronbach’s alpha	0.87			0.80	0.85	0.63		

a Not included in the final version of the scale.

Based on the results from EFA, we decided to retain the one-factor solution as the best candidate to represent the relations between items, to be used in further analyses.

### Confirmatory factor analysis

We tested the model derived from EFA by means of CFA, using the second randomly selected sub-sample (*N*=235). Results showed that the model fit to the data was not satisfactory (CFI = 0.92, RMSEA = 0.23 with 90% CI = 0.21–0.26, SRMR = 0.12). We inspected the modification indices derived from the model and we noticed that three items (Item 2, Item 4, and Item 8) were major contributors to model misfit. We then re-tested the model after dropping those items and we found a substantial improvement in model fit (CFI = 1.00, RMSEA = 0.06 with 90% CI = 0.00–0.11, SRMR = 0.02). Regarding reliability, we found that the model showed a satisfactory value of Omega (0.87). Based on those results, we decided to retain the 6-item model for further analyses.

### Item response theory

We fit and evaluated two alternative GRM models, based on different assumptions: (i) a model in which all items are equally discriminating between respondents and (ii) a model in which discrimination parameters are constrained to be equal across items. Then, we compared their fit, aiming to identify the best candidate to represent the data (Rizopoulos, 2006). Results showed that the unconstrained model (AIC = 3134.44, BIC = 3238.23, logLik = −1537.22, marginal reliability = 0.90) performed significantly better (*p* < 0.001) than the constrained model (AIC = 3165.16, BIC=3251.65, logLik = −1557.58, marginal reliability = 0.89). Therefore we decided to use the former for further inspection.

The residual correlation matrix extracted from the unconstrained model showed an absolute average residual correlation of 0.01, with 6 item pairs showing negative residual correlations (<0.21), suggesting issues of local dependence. The inspection of item parameters showed that Item 1 had the highest discrimination (*α* = 4.31), followed by Item 9 (*α* = 3.21), whereas Item 7 (*α* = 1.81), and Item 3 (*α* = 1.62) were the least discriminating items (Table 4).

**Table 4.** CORAS graded response model, standardised item parameters’ estimates and errors (*N* = 235).

Item number	*α*	*SE*	*β*1	*SE*	*β*2	*SE*	*β*3	*SE*	*β*4	*SE*
1. Gut feeling of own likelihood of infection	4.31	0.70	6.68	1.02	0.40	0.37	6.41	0.97	−8.99	1.30
2. Can picture self catching it	1.62	0.20	3.20	0.32	1.07	0.20	−1.80	0.23	−3.82	0.37
3. Sure I will not be infected	2.22	0.26	5.57	0.60	2.40	0.29	0.43	0.22	−1.73	0.26
4. Unlikely to get infected	2.75	0.33	5.20	0.57	1.02	0.27	−0.97	0.27	−3.73	0.42
5. Feel vulnerable	1.81	0.22	2.80	0.30	0.02	0.20	−1.42	0.22	−3.99	0.39
6. Self-rated chance of infection	3.21	0.47	9.43	1.39	4.72	0.64	−2.71	0.43	−5.55	0.68

**Figure 2.**
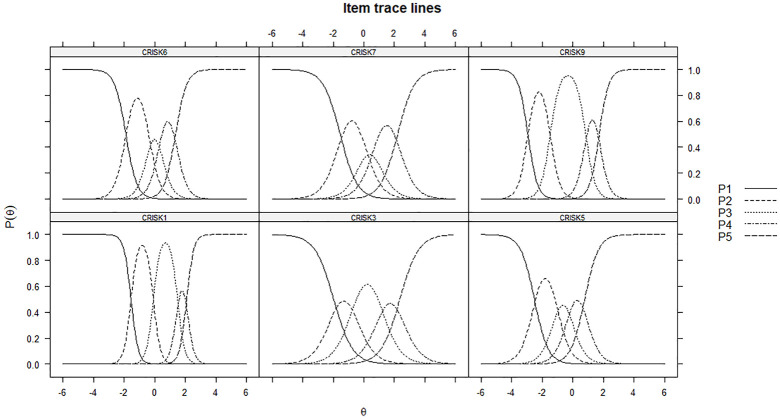
GRM, item response categories curves.

We examined IRCCs by plotting the probabilities of response categories (1–5) to be endorsed at different levels of respondents’ perceived risk (Figure 2), for all items. Best performing items are those whose IRCCs shows a wide range of probabilities across all levels of theta, indicating adequate targeting of respondents across different degrees of perceived risk. All items performed reasonably well, with response categories peaking and dispersing in an orderly fashion, indicating satisfactory targeting of respondents with different levels of perceived risk. However, Item 7 showed that the probability to endorse category 3 at theta = 0 was about 30%, not much higher than the probability to endorse category 2, suggesting the opportunity to collapse the two.

**Figure 3.**
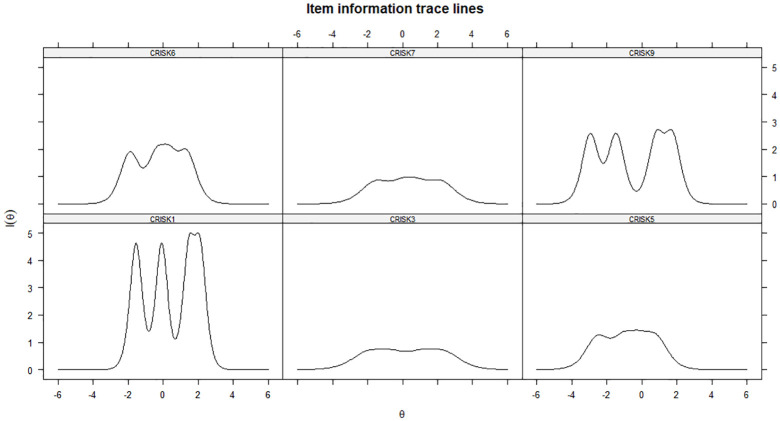
GRM, item information curves.

**Figure 4.**
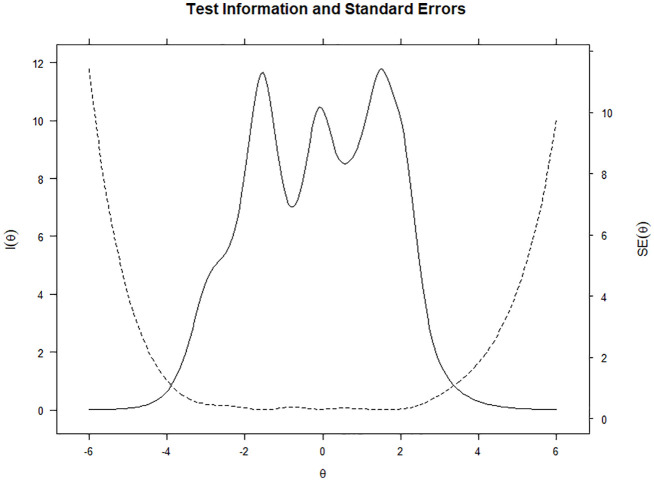
GRM, test information curve and standard error.

We plotted IIFs to assess items’ discrimination as a function of perceived risk of COVID-19. Item 1 provided the highest degree of information across the continuum of theta, followed by Item 6 and Item 9, whereas Item 7 and Item 3 provided the least amount of statistical information (Figure 3). Figure 4 shows the total test information curve and standard error of measurement.

Last, we tested for differential item functioning by gender, age, and ethnicity on all the CORAS items. We first recoded gender by dropping observations obtained with participants who self-reported their gender as non-binary, due to a too low number of observations (*N* = 2), and age, dividing observations into two groups representing participants younger than 30 years versus participants aged 30 years or older, respectively. Main ethnicity included two categories, that is, participants who self-reported White British ethnicity and those who reported being of a BAME background, respectively. The results showed no differential item functioning on any of the CORAS items, with no significant difference found in either *χ*^2^ probability, pseudo *R*^2^ change, or proportional *β* level change, suggesting item invariance across groups for all items.

### Convergent and criterion validity

We tested for the validity of the CORAS on the whole sample (*N* = 470), computing total scores by summing up participants’ responses on all the scales’ items. We then estimated Spearman’s rho correlations between the CORAS and the Fear of COVID-19 Scale, for convergent validity, and the CORAS and the COVID-19 Preventive Behaviours Index for criterion validity, respectively. We found a significant and positive (Spearman’s rho = 0.54, *p* < 0.001) correlation in the first case, confirming the convergent validity of the CORAS, and a significant and positive, although lower correlation in the second case (Spearman’s rho = 0.21, *p* < 0.001).

## Discussion

The aim of this study was to develop, test, and validate the factor structure and psychometric properties of the COVID-19 Own Risk Appraisal Scale (CORAS), a tool for the measurement of perceived personal risk, in two participant samples from the United Kingdom. We used exploratory factor analysis and confirmatory factor analysis to explore and test a theoretical model based on the nine items of the CORAS loading onto one factor of perceived personal risk of infection. Results indicated that a one-factor 6-item model may be the best to represent the data. Overall, the model achieved good fit. IRT analysis showed that items provided a range of discrimination across the levels of theta, thus discriminating respondents with different levels of perceived risk. The model fits the data well, showing satisfactory reliability and no significant differential item functioning by age, gender, and ethnicity. Regarding validity, the CORAS showed high positive correlations with the Fear of COVID-19 Scale (Ahorsu et al., 2020) and positive correlations with the COVID-19 Preventive Behaviours Index (Breakwell et al., 2020).

Based on the findings reported here, the CORAS represents a reliable and valid measure of perceived likelihood of personally becoming infected. It incorporates items that allow for the likelihood estimate to be based explicitly on intuition (e.g. having a ‘gut feeling’ about one’s vulnerability to the disease or being able to ‘picture’ oneself with the disease) as well as any considered analysis of available evidence. In total, three items were removed from the original 9-item measure. Item 8 had to be removed from the initial nine used because responses on it did not differentiate between individuals. In retrospect, this item (‘There is a chance, no matter how small, I could get COVID-19’) was very likely to have elicited consensual agreement. Although the items originally proposed for CORAS contained one tapping into the emotional reaction to the disease (e.g. worry about contracting COVID-19), the results from CFA indicated that this item (2) had a poor fit to the model, with its residual correlating with other items’ residuals, which resulted in its removal from the scale. This supports the argument that emotional reactions to the likelihood of contracting the disease need to be measured independently from perceived likelihood itself. Finally, Item 4 (‘Getting COVID-19 is something I am concerned about’) which similarly focussed on an affective response to the prospect of contracting COVID-19 rather than to an estimate of the likelihood of this happening also had a poor fit to the model, requiring deletion.

The CORAS is a brief, reliable and valid measure of perceived likelihood of own COVID-19 infection. It is useful in assessing changes over time in public perceptions of personal risk. Our data emphasise that fear and perceived likelihood of own infection should be treated as separate but complementary predictors of COVID-19 preventive behaviours. In terms of health education messaging this is worth considering further. Messaging that does not overly stimulate fearfulness but does influence intuitive and analytic appraisals of personal likelihood of infection may be important (Witte and Allen, 2000). For instance, a well-researched bias in risk perception involving feelings of personal invulnerability is found to intensify or justify risk-taking (Hill et al., 2012). In the context of COVID-19, this bias, which is more likely to occur in younger age groups, may be affirmed or accentuated by the consequence patterns of the disease (young people being less likely to suffer extreme, dangerous symptoms even though they are just as likely to become infected). If this intuitive but biased appraisal of likelihood of infection becomes common in younger age groups and they ignore self-protection advice, attempts to limit the spread of the disease will be undermined. Crafting health protection advice that acknowledges intuitive appraisals of risk will result in more targeting of messages. For that to occur it is important for own risk appraisals to be monitored. CORAS can help in that monitoring. Given the focus on one’s *own* risk of infection in the CORAS, this tool may be especially important in predicting behaviours intended to reduce one’s risk of the disease.

### Future directions

A convenience sampling approach was used in this study to test and validate the CORAS. Future research should aim to recruit a more representative sample from the UK population. Also, the CORAS was tested only in participants who use the Internet and, thus, future research should use other sampling approaches which might allow greater access to other groups at high risk of COVID-19 but who are less likely to be recruited online, such as the elderly. Furthermore, participants were recruited only in the UK where disease incidence and the mortality rate have been high. Risk appraisal may be different in other countries, suggesting the CORAS should also be validated in non-UK samples. The simplicity of the scale will be likely to make it transferable to other populations and contexts.

## Conclusion

This article summarises the development, validation, and psychometric testing of the COVID-19 Own Risk Appraisal Scale (CORAS), which is a novel six-item tool for the measurement of perceived personal risk of exposure to COVID-19. It exhibits good reliability and good concurrent validity with related constructs, namely fear of COVID-19 and engagement in COVID-19 preventive behaviours. Most existing instruments either rely on single-item measures or conflate the dimensions of emotional versus cognitive risk and personal risk versus risk to others. This limits their value. Conversely, the CORAS is a relatively short tool for measuring the cognitive and intuitive aspects of risk perception, providing a pragmatic and robust measurement index for use in empirical research into risk and behaviour in relation to the pandemic.

## Appendix

### The COVID-19 Own Risk Appraisal Scale (CORAS)

1. What is your gut feeling about how likely you are to get infected with COVID-19? (1 = extremely unlikely; 5 = extremely likely).
2. Picturing myself getting COVID-19 is something I find: (1 = very hard to do; 5 = extremely easy to do).
3. I am sure I will NOT get infected with COVID-19* (1 = strongly disagree; 5 = strongly agree).
4. I feel I am unlikely to get infected with COVID-19* (1 = strongly disagree; 5 = strongly agree).
5. I feel vulnerable to COVID-19 infection (1 = strongly disagree; 5 = strongly agree).
6. I think my chances of getting infected with COVID-19 are: (1 = zero; 5 = very large)
  *reverse-scored
